# Vitamin D-Binding Protein Levels in Plasma and Gingival Crevicular Fluid of Patients with Generalized Aggressive Periodontitis

**DOI:** 10.1155/2014/783575

**Published:** 2014-05-12

**Authors:** Xin Zhang, Huanxin Meng, Li Xu, Li Zhang, Dong Shi, Xianghui Feng, Ruifang Lu, Zhibin Chen

**Affiliations:** ^1^Department of Periodontology, Peking University School and Hospital of Stomatology, 22 Zhongguancun Nandajie, Haidian District, Beijing 100081, China; ^2^Department of Stomatology, Peking Union Medical College Hospital, Beijing 100730, China

## Abstract

Vitamin D-binding protein (DBP) is the main transport protein of vitamin D and plays an important role in the immune system and host defenses. The purpose of this study was to measure DBP levels in plasma and gingival crevicular fluid (GCF) of patients with generalized aggressive periodontitis (GAgP), in comparison to healthy controls, with the goal of elucidating the relationship between DBP and GAgP. Fifty-nine GAgP patients and 58 healthy controls were recruited for the study; clinical parameters of probing depths (PD), bleeding index, and attachment loss (AL) were recorded. DBP levels were measured by enzyme-linked immunosorbent assay. From the results, GAgP patients had higher plasma DBP concentrations (*P* < 0.001) but lower GCF DBP concentrations (*P* < 0.001) than healthy controls. In GAgP group, after controlling the potential confounders of age, gender, smoking status, and BMI index, GCF DBP concentrations correlated negatively with PD (*P* < 0.001) and AL (*P* = 0.009). Within the limits of the study, we concluded that decreased GCF DBP level and increased plasma DBP level are associated with periodontitis.

## 1. Introduction

Periodontitis is primarily a bacterial infection caused by a diverse group of microorganisms [1]. Generalized aggressive periodontitis (GAgP) is a subtype of periodontitis that mainly affects younger patients and is characterized by episodic and rapid loss of periodontal supporting tissues [2]. Though microorganisms are considered to be the etiologic agent that causes this inflammatory lesion, it is the chemical mediators of inflammation that play a pivotal role in the loss of periodontal connective tissue, as well as supporting alveolar bone [3].

Vitamin D has been shown to have immunomodulatory effects in vitro and in animal studies. Previous research from our group revealed a relationship between 25-hydroxy-vitamin D (25[OH]D) and GAgP. Specifically, we reported that plasma 25(OH)D levels were higher in patients with GAgP, as compared to healthy controls, and were positively correlated with an index of gingival bleeding [4]. It has also been shown that initial periodontal therapy reduces both local and systemic 25(OH)D levels [5]. Furthermore, polymorphisms in the gene encoding the vitamin D receptor have been reported in several studies to be associated with periodontitis [6–10]. Taken together, these studies suggest a potential role for the vitamin D pathway in maintaining periodontal health.

Vitamin D-binding protein (DBP), also known as group-specific component (Gc), is another important element in vitamin D pathways. It is the main transport protein of vitamin D in circulation and plays an important part in vitamin D bioavailability. Besides, it also has anti-inflammatory and immunomodulatory functions independent of vitamin D carriage, such as coactivating macrophages, enhancing the chemotactic activity of C5-derived peptides, and associating with many immune cell surfaces including neutrophils. In addition it scavenges actins released from necrotic cells, binds fatty acids, and plays a part in bone modulation [11, 12]. The diverse roles of DBP, including its relationship with vitamin D levels, indicate its potential importance in a range of diseases. For example, DBP was found in the airways of patients with chronic obstructive pulmonary disease and acute respiratory distress syndrome and is believed to stimulate the monocyte inflammatory response in the lung by enhancing monocyte responses to C5-derived peptides [13]. In terms of cardiovascular disease, high expression of DBP protein has been found in fresh thrombotic plaques and in the serum of patients with ST elevation myocardial infarction [14]. Other disease states associated with altered DBP levels include tuberculosis, type 1 diabetes, sepsis, and cystic fibrosis [15–18].

With regard to periodontitis, there are few previous studies which have studied DBP levels in relation to this disease. A relatively small study (1987) reported elevated DBP concentrations in whole saliva of patients with periodontitis, as compared to dentulous or edentulous control subjects, and positively correlated DBP concentration with Gingival Index scores [19]. More recently, Wu et al. compared the proteomic profile of whole unstimulated saliva in GAgP patients with those of healthy controls and confirmed the rise of saliva DBP levels in periodontitis [20]. Our previous study firstly determined higher plasma DBP levels in patients with GAgP and found that the levels were associated with inflammatory cell types and inflammatory markers [21]. The present study was to assess DBP levels in GCF as well as plasma of patients with GAgP in comparison to healthy controls and to find out the link between DBP and GAgP.

## 2. Materials and Methods

### 2.1. Study Population

Fifty-nine GAgP patients were recruited from the clinic of the Department of Periodontology at Peking University Hospital of Stomatology from July 2001 to October 2007. Diagnostic criteria for GAgP were defined according to the classification proposed at the International Workshop for the Classification of Periodontal Diseases and Conditions in 1999. Specifically, (1) the onset of the periodontal disease occurred under 35 years of age; (2) at least eight teeth had a probing depth (PD) over 6 mm and at least three of them were not first molars or incisors; and (3) radiographic evidence of alveolar bone loss was present. Fifty-eight periodontal healthy controls were selected from staff and students at the School of Stomatology; none of them had any clinical evidence of periodontitis (PD ≤ 3 mm; the percentage of sites with bleeding on probing (BOP) was <10%; no attachment loss (AL) and no bone loss visible on radiographs). Subjects with systemic disease, who had periodontal therapy within the previous year, who received antibiotics within the previous 3 months, were pregnant or were receiving vitamin D or calcium supplements (e.g., vitamin D, calcium carbonate, calcium lactate, or calcium gluconate) were excluded from the study. Each subject in the study completed a questionnaire at the beginning of the study, and their age, height, weight, body mass index (BMI), and smoking status were recorded. Smokers were defined as subjects who were currently smoking (smoked routinely and never stopped or tried to stop smoking in recent three months) and nonsmokers were defined as subjects who had never smoked or had ceased smoking more than one year. The study was conducted with informed consent of all subjects and approved by the Ethics Committee of Peking University Health Science Center.

### 2.2. Clinical Examination

A full-mouth periodontal examination of each subject was conducted using a William's periodontal probe. PD and AL were recorded for each tooth at six sites (mesial, distal, and middle sites of facial and lingual sides), except wisdom teeth. AL was measured as the distance between the cementoenamel junction and the bottom of the periodontal pocket. Bleeding index (BI) [22] was also recorded for each tooth. Sites with PD over 6 mm and AL over 5 mm were defined as sites of severe periodontitis. The mean PD, AL, and BI and the percentage of severe sites were calculated for each subject. All examinations were performed by two experienced practitioners. In addition to the dental exam, each patient had a set of full-mouth periapical radiographs taken.

### 2.3. Sample Collection and Processing

The blood samples were obtained from each subject by standard venipuncture using EDTA-containing collection tubes; all blood samples were collected between 8:00 and 10:00 am. Plasma was separated by centrifugation and immediately stored at −70°C until the time of the DBP assay. GCF was collected at two sites from 22 GAgP patients (seven males and 15 females) and 23 healthy controls (nine males and 14 females). The GCF collection sites included one around the right maxillary incisor and the other in the region of left mandibular molar. In GAgP patients, all of these sites were affected by periodontitis, with PD over 4 mm and AL over 1 mm. The method of GCF collection and volume determination were performed as previously described [5]. Briefly, the test site was air-dried and isolated with a roll of cotton, and supragingival plaque was removed without touching the marginal gingiva. A paper strip (Whatman, Maidstone, UK) was then inserted into the respective periodontal pocket until mild resistance was felt and was then left in place for 30 seconds. Strips with any visible contamination of blood were discarded. Before sampling, each strip was placed into a sterile 0.5 mL Eppendorf tube and weighed using an electronic scale (AE240S, Mettler, Zurich, Switzerland). After GCF collection, the strip was placed into the same tube and reweighed within 30 minutes of collection. The tube containing the strip was then stored at −70°C until the time of the DBP assay. The difference between the weight of the tube and strip before and after collection was used to calculate the volume of GCF collected.

GCF is considered to be a serum exudate, as such we established a standard curve for GCF volume with GCF weight using human serum. Specifically, 0.1, 0.3, 0.5, 1.0, 1.5, and 2.5 *μ*L of human serum were dropped onto individual paper strips. The difference in the weight of each strip before and after addition of a defined drop of human serum was used to calculate the weight of serum added. A linear regression model between weight and volume was then established (*r* ^2^ = 0.993; *P* < 0.001).

### 2.4. DBP Assay

At the time of the DBP assay, the 0.5 mL Eppendorf tubes containing GCF-saturated strips were thawed out at room temperature and a 100 *μ*L of phosphate buffered solution (pH 7.4) was added to each tube. The tubes were gently shaken at 4°C for 20 minutes and then centrifuged at 13,000 rpm (rotor radius = 5.5 cm) for 10 minutes. The DBP levels in the GCF supernatant and plasma were measured using a commercially available enzyme-linked immunosorbent assay kit (BioSource Systems, Invitrogen, Grand Island, NY, USA). These assays were performed according to the manufacturer's protocol. The total GCF DBP level was calculated according to the measured DBP concentration. Calculation of DBP concentration in each GCF sample was performed by dividing the amount of DBP by the volume of GCF.

### 2.5. Statistical Analysis

Both clinical and biochemical parameters were reported as mean ± standard deviation. Variables were tested for normality using the Shapiro-Wilk test. As they were not normally distributed, the data were analyzed using nonparametric tests. The levels of DBP were compared between GAgP group and control group using the Mann-Whitney *U*-test. Partial correlations between DBP levels and periodontal clinical parameters were analyzed with adjustment for age, gender, smoking status, and BMI index. Correlations between GCF DBP levels and clinical parameters were analyzed on site-level data, and clinical parameters of BI, PD, and AL of the sample site were used. While analysis about correlations between plasma DBP levels and clinical parameters was based on subject-level data, the calculated clinical parameters of mean BI, PD, and AL and percentage of severe sites for each subject were used. Statistical analyses were carried out using SPSS software, version 11.5 (SPSS Inc, Chicago, IL, USA), and *P* values <0.05 were considered statistically significant.

## 3. Results

**Figure 1:**
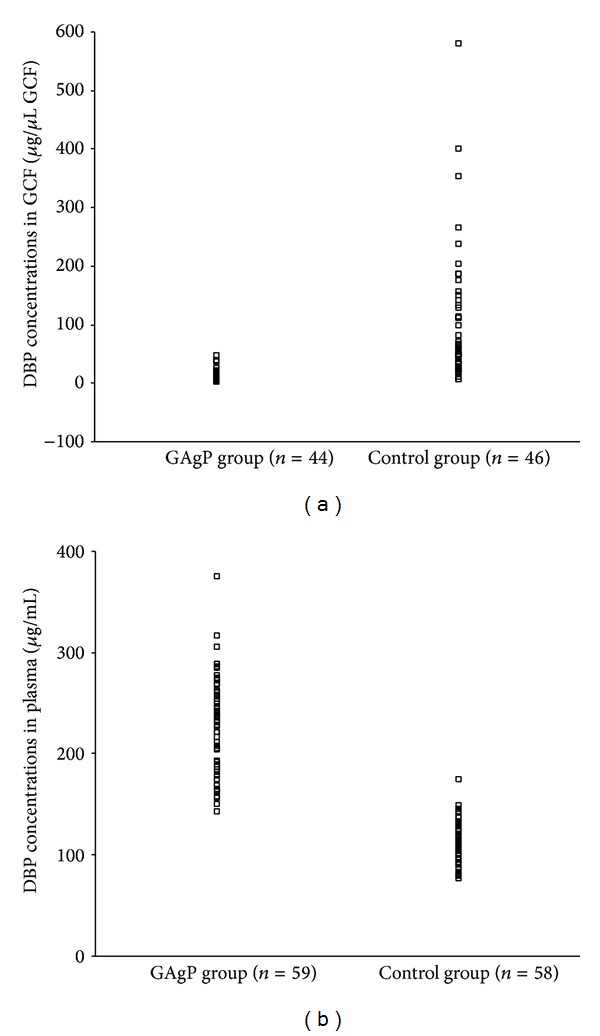
(a) DBP concentrations in GCF of GAgP group were significantly lower than in control group (*P* < 0.001); (b) DBP concentrations in plasma of GAgP group were significantly higher than in control group (*P* < 0.001).

The basic clinical characteristics of the GAgP and healthy control groups are summarized in Table 1. The two groups were not different with respect to age, gender, smoking status, and BMI index. As expected, generally more severe clinical indices of periodontal disease were observed in the GAgP group. The GCF data was presented on site level. The mean PD of the 44 periodontitis sites selected for GCF sampling was 6.70 ± 1.89 mm, with a range from 4 mm to 10 mm. The mean AL of these sites was 6.39 ± 2.52 mm, with a range from 1 mm to 13 mm. As presented in Table 2 and Figure 1, the GAgP group had significantly lower GCF DBP concentrations (13.39 ± 10.20 *μ*g/*μ*L GCF versus 166.01 ± 298.12 *μ*g/*μ*L GCF; *P* < 0.001) and higher plasma DBP concentrations (231.75 ± 47.86 *μ*g/mL versus 111.12 ± 20.97 *μ*g/mL; *P* < 0.001) compared to the healthy control group. In GAgP group, after controlling the potential confounders of age, gender, BMI, and smoking status, GCF DBP concentration correlated negatively with PD (*r* = −0.504, *P* = 0.001) and AL (*r* = −0.410, *P* = 0.009) of the sample site (Table 3). No correlations were found between plasma DBP concentration and periodontal clinical parameters in neither GAgP group nor control group as shown in Table 4.

## 4. Discussion

DBP is a plasma a2-globulin with a molecular weight of 52–59 kDa and is recognized as a member of a multigene family that includes albumin, *α*-fetoprotein, and *α*-albumin/afamin. The DBP gene is expressed in a wide variety of tissues, but the vast majority of serum DBP is derived from gene expression and secretion by the liver. It is abundant in plasma and exerts multiple biological functions, which range from transporting vitamin D metabolites, scavenging actins, to recently most discussed roles in immune system and host defense [11].

DBP was implied to be a normal constituent of parotid saliva and its levels in parotid saliva did not vary according to the presence or absence of periodontal disease and were irrespective of the presence or absence of the teeth [19]. While DBP concentrations in whole saliva of patients with periodontitis were increased compared to healthy controls and correlated positively with periodontal inflammation, the elevated DBP levels in whole saliva were reported less than 1 *μ*g/mL, which were far lower than those in GCF according to our reports and were proposed to come from GCF. It is reasonable considering the far differences between GCF and saliva DBP levels, and the fact that, in cases of periodontitis, both GCF volume and flow rate were increased significantly [23]. It was much easier for the high GCF DBP to flow into the mouth and finally contributed to the increased saliva DBP levels.

On healthy conditions, DBP presences in GCF were abundant and its levels were even higher than those in plasma. It is indicated that DBP might exert a role in periodontal health and that periodontal tissues might be another sources of GCF DBP other than serum. The significant decrease of GCF DBP levels in periodontitis patients might be a consequence of a lack of effective production or an increase of local consumption. For more information, it might be necessary to investigate the distributions or expressions of DBP in healthy and periodontitis tissues. Also, longitudinal study about how periodontal therapy influences local and plasma DBP levels is essential to confirm the relationship between DBP and periodontal inflammation. Nevertheless our data indicate that DBP is present in the GCF and available to participate in the inflammatory response to periodontal challenge.

DBP is the main transport protein of vitamin D in the circulation, and plasma 25(OH)D levels have been reported to be increased in patients with GAgP, as well as being associated with periodontal inflammation [4, 5]. In concordance with these previous studies, we observed that plasma DBP levels of patients with GAgP were higher than controls; however, no correlation between plasma DBP concentrations and the previously measured plasma 25(OH)D levels in the same 44 GAgP patients (data not shown) was found. We interpret this result to indicate that the increased DBP concentration might not be a direct response to a change in vitamin D status. Mechanisms independent of vitamin D levels might explain the rise in plasma DBP level seen in GAgP patients and also in the potential role of DBP in periodontitis. This finding is in agreement with a previous study in patients with type 1 diabetes, where altered DBP concentrations were not related to 25(OH)D levels [15].

In the current study, the plasma concentrations of DBP in healthy individual were assayed to be 77.51–175.10 *μ*g/mL, which were lower than those reported in the other race [11, 15]. It is worth noting that plasma 25(OH)D levels of Chinese people also seemed a little lower than in other people [4]. So one reason might be the differences caused by the human race. Besides the differences in the sensitivity of ELISA kits might make the data provided not always comparable.

DBP is implied to be an acute phase reactant produced by liver. Its hepatic synthesis could be upregulated by proinflammatory cytokines such as interleukin-6, and it is known that plasma interleukin-6 levels are increased in cases of GAgP, thus such a proinflammatory response may contribute to the increased DBP levels observed in patients with GAgP [24, 25]. In addition to the liver, several other tissues express DBP, including the kidney, adipose tissue, and gonads; however, it is not known whether DBP expression in these tissues can be regulated by the immune system [26]. In direct relation to cells of the immune system, it is known that DBP mRNA is expressed in activated monocytes [27]; it can be secreted by neutrophils and this level of secretion was enhanced after C5a stimulation [28]; it binds to the surface of neutrophils which is essential for DBP's chemotactic activity [29]; and neutrophils activated by LPS have an increased number of DBP-binding sites [30]. Taken together, these studies suggest that, in cases of periodontitis, increased numbers of neutrophils with an enhanced ability to secrete and bind DBP might also contribute to systemic increases in DBP levels.

From the results, GAgP patients have higher plasma DBP levels but lower GCF DBP levels when compared to healthy controls. But no significant correlations were found between these two presences in neither GAgP patients nor healthy controls, when taking the average of the two data as the GCF DBP levels for those 22 GAgP patients and 23 healthy controls (data not shown). According to the methods and materials, two sites of the individuals were chosen to collect GCF samples. These two sites were not always the most severe site of the patients with GAgP; on the other hand, the sampling sites were just two and may not represent the whole periodontal inflammation of the individual. Further research is needed to gain more detailed information about the potential roles of DBP in the pathogenesis of periodontitis.

## 5. Conclusions

In summary, DBP was measured in the GCF and plasma of patients with GAgP. In comparison to healthy controls, we found that patients with GAgP had higher plasma levels but lower GCF DBP concentrations. The greater the periodontal destruction is, the lesser the GCF DBP concentration is. Further research is needed to investigate the expressions of DBP in periodontium of periodontal health and disease and to study how periodontal therapy influences systemic and local DBP levels.

## Figures and Tables

**Table 1:** Basic clinical characteristics of GAgP patients and healthy controls.

	GAgP group (*n* = 59)	Control group (*n* = 58)
Age (years)	26.7 ± 4.9	25.4 ± 3.6
Gender (*n*; male/female)	20/39	25/33
BMI (kg/m^2^)	21.58 ± 3.43	21.22 ± 2.04
Smoking (*n*; smoker/nonsmoker)	5/54	1/57
Mean BI	3.69 ± 0.33*	1.13 ± 0.15
Mean PD (mm)	4.81 ± 0.99*	1.63 ± 0.35
Mean AL (mm)	4.75 ± 1.84*	0
Severe site % (% of sites)	32.14 ± 21.09*	0

Data are presented as mean ± SD or number of subjects as indicated; *P < 0.05, compared to healthy controls.

**Table 2:** Comparisons of GCF DBP levels and plasma DBP levels between GAgP patients and healthy controls.

DBP levels	GAgP group	Control group	*P* value
	Mean ± SD	Mean ± SD	
GCF DBP (*μ*g/*μ*L GCF)	13.39 ± 10.20	166.01 ± 298.12	<0.001
	*n* = 44	*n* = 46	
Plasma DBP (*μ*g/mL)	231.75 ± 47.86	111.12 ± 20.97	<0.001
	*n* = 59	*n* = 58	

**Table 3:** Partial correlations between GCF DBP concentrations and clinical parameters after adjusting for age, gender, smoking status, and BMI index.

GCF DBP (μg/μL GCF)	GAgP group (n = 44)		Control group (n = 46)	
	*r*	*P* value	*r*	*P* value
BI	−0.481	0.768	None	—
PD	−0.504	0.001	None	—
AL	−0.410	0.009	None	—

**Table 4:** Partial correlations between plasma DBP concentrations and clinical parameters after adjusting for age, gender, smoking status, and BMI index.

plasma DBP (*μ*g/mL)	GAgP group (*n* = 59)		Control group (*n* = 58)	
	*r *	*P* value	*r *	*P* value
Mean BI	0.1588	0.251	0.048	0.768
Mean PD	−0.1347	0.327	−0.190	0.216
Mean AL	−0.164	0.230	None	—
Severe sites %	−0.167	0.233	None	—
